# Spatial ecology of translocated raccoons

**DOI:** 10.1038/s41598-023-37323-6

**Published:** 2023-06-27

**Authors:** Jacob E. Hill, James L. Helton, Richard B. Chipman, Amy T. Gilbert, James C. Beasley, Guha Dharmarajan, Olin E. Rhodes

**Affiliations:** 1 Savannah River Ecology Laboratory, University of Georgia, PO Drawer E, Aiken, SC 29802 USA; 2 Warnell School of Forestry and Natural Resources, University of Georgia, 180 E Green St, Athens, GA 30602 USA; 3 National Rabies Management Program, USDA, APHIS, Wildlife Services, Concord, NH 03301 USA; 4 National Wildlife Research Center, USDA, APHIS, Wildlife Services, 4101 Laporte Ave, Fort Collins, CO 80521 USA; 5 Odum School of Ecology, University of Georgia, 140 E Green St, Athens, GA 30602 USA

**Keywords:** Behavioural ecology, Ecosystem ecology

## Abstract

Raccoons (*Procyon lotor*) are routinely translocated both legally and illegally to mitigate conflicts with humans, which has contributed to the spread of rabies virus across eastern North America. The movement behavior of translocated raccoons has important ramifications for disease transmission yet remains understudied and poorly quantified. To examine the spatial ecology of raccoons following experimental translocation, we performed reciprocal 16 km-distance translocations of 30 raccoons between habitats of high and low raccoon density (bottomland hardwood and upland pine, respectively) across the Savannah River Site (SRS) in Aiken, South Carolina, USA (2018–2019). Translocation influenced patterns of raccoon space use, with translocated animals exhibiting a 13-fold increase in 95% utilization distributions (UDs) post- compared to pre-translocation (mean 95% UD 35.8 ± 36.1 km^2^ vs 1.96 ± 1.17 km^2^). Raccoons originating from upland pine habitats consistently had greater space use and larger nightly movement distances post-translocation compared to raccoons moved from bottomland hardwood habitats, whereas these differences were generally not observed prior to translocation. Estimated home ranges of male raccoons were twice the area as estimated for female raccoons, on average, and this pattern was not affected by translocation. After a transient period lasting on average 36.5 days (SD = 30.0, range = 3.25–92.8), raccoons often resumed pre-experiment movement behavior, with 95% UD sizes not different from those prior to translocation (mean = 2.27 ± 1.63km^2^). Most animals established new home ranges after translocation, whereas three raccoons moved > 16 km from their release point back to the original capture location. Four animals crossed a 100-m wide river within the SRS post-translocation, but this behavior was not documented among collared raccoons prior to translocation. Large increases in space use combined with the crossing of geographic barriers such as rivers may lead to elevated contact rates with conspecifics, which can heighten disease transmission risks following translocation. These results provide additional insights regarding the potential impacts of raccoon translocation towards population level risks of rabies outbreaks and underscore the need to discourage mesocarnivore translocations to prevent further spread of wildlife rabies.

## Introduction

Wildlife translocation, defined as “the transport and release of wild animals from one location to another with emphasis on nuisance and damage”^1^ may be used to achieve a variety of management objectives, but can have unanticipated consequences by facilitating disease spread^2^. Translocations of raccoons (*Procyon lotor*), for example, led to epizootic spread of rabies virus (RABV) throughout populations in eastern North America^3^. Previously limited to the southeastern United States, the spread of raccoon RABV variant into much of the mid-Atlantic and northeastern United States and Canada was facilitated by the translocation of rabid raccoons from Florida to West Virginia for hunting purposes^3,4^. Subsequent translocations may also be responsible for further spread of raccoon RABV in the US and Canada^5,6^. The translocation of raccoons and other mesocarnivores has directly resulted in the expansion of the geographic extent of raccoon RABV in North America, which encompasses most of the region east of the Appalachian Mountains, spanning from Florida to Maine^7^. Given historical and contemporary disease risks recognized with translocation of mesocarnivores, the practice is discouraged as part of comprehensive guidelines for the prevention and control of rabies in wild animals in the United States^8^.

An important management strategy for wildlife RABV control includes the coordinated distribution of oral rabies vaccine in both rural and developed habitats to prevent the spread of and locally eliminate specific RABV variants in mesocarnivore populations^9^. Threats to human and animal health led to the implementation of local and national wildlife management programs using oral rabies vaccination (ORV) as the primary method for landscape level wildlife rabies control^3,10^. Millions of vaccine baits are distributed each year by aerial methods such as planes and helicopters, as well as ground means such as vehicles and bait stations, targeting raccoons, striped skunks (*Mephitis mephitis*), gray and red foxes (*Urocyon cinereoargenteus* and *Vulpes vulpes*) and coyotes (*Canis latrans*) to establish zones with sufficient population immunity to prevent, reduce or eliminate the incidence of specific RABV variants^7^. As a result of ORV programs, the dog-coyote and Texas gray fox RABV variants have been eliminated from coyote and gray fox populations in Texas, respectively, and cases of raccoon RABV have been geographically contained in the eastern US^11,12^. The effectiveness of this management approach requires a significant long-term investment of public resources^13^, where financial investments and management objectives can be jeopardized by translocation that can compromise rabies control and result in costly contingency actions^9,14^.

Despite the potential impacts to wildlife rabies management programs, there is a continuous risk of raccoon and other mesocarnivore translocation. Raccoons are ecological generalists and abundant in a variety of habitats. Raccoon population densities can be significantly higher in more developed landscapes due to the increased availability of anthropogenic resources including food and preferred habitat for denning^15–17^. Raccoons are the primary source of nuisance wildlife complaints in many areas^18–20^. Although many states now prohibit the translocation of raccoons and other rabies reservoir species, higher rates of human-wildlife conflict combined with general societal concerns regarding lethal management in some areas of the country may lead to illegal movement of raccoons^21,22^. Additionally, the propensity for raccoons to forage in unsecured dumpsters may result in unintentional relocation in garbage trucks to distant landfills or trash transfer stations^2^.

The movement behavior of translocated raccoons has important implications for rabies management yet remains understudied. One simulation study of non-translocated raccoons demonstrated that larger and more varied home ranges correlated with increased rabies transmission risk^23^. Translocation for many mammals, including raccoons, is often followed by an exploratory period in which animals exhibit extensive movements as they attempt to navigate back to their original habitat or locate resources in their novel habitat^24–26^. Such exploration may also lead translocated animals to exhibit atypical movement behavior, such as crossing water bodies^27^. The movement behavior of translocated animals may be markedly different from that of resident animals and is typically characterized by higher movement rates and larger home ranges^24^.

Several factors contribute to variation in space use among translocated animals. Translocations of several mammal species have demonstrated that sex-specific differences in movement may persist after translocation due to disparities in resource requirements or habitat selection^28–30^. Considering that male raccoons generally have larger home ranges than females^31–33^, male raccoons could be expected to maintain larger home ranges than female raccoons after translocation. The density of conspecifics in the destination habitat may further influence movement. Translocated pine martens (*Martes martes*), for example, moved further from their release point when the destination habitat had higher conspecific abundance, likely due to greater resource competition^30^. Similarly, wild boar (*Sus scrofa*) translocated to habitats with higher population density had much smaller exploratory movements and exploratory ranges than individuals translocated to habitats with lower population density^26^.

Additionally, space and habitat use is determined in part by prior use patterns^34,35^. Many mammals exhibit density-dependent home ranges in which greater densities of individuals resulting from higher resource availability lead to smaller home ranges^36,37^, and these previous patterns in home range size may carry over after translocation. Additionally, animals from high-density habitats may have experienced increased rates of conspecific encounters prior to translocation and be less likely to avoid other raccoons in the destination habitat. This may result in smaller home ranges because they compete directly with conspecifics for resources. Conversely, animals from low-density habitats may be more likely to avoid conspecifics due to less frequent interspecific interaction, resulting in wider ranging behavior as they seek to avoid other raccoons. However, this pattern may not occur if resource abundance and distribution is more important than social interactions as raccoons may congregate when resources are readily available^15,38^.

To provide further insight into the movement behavior of translocated racoons and the potential associated risk of rabies transmission, we performed reciprocal translocations of male and female raccoons between habitats of high and low raccoon densities. We tested the hypothesis that translocation influenced movement behavior, predicting that translocated raccoons would have larger home ranges than control (i.e. non-translocated) animals. Additionally, we tested the hypothesis that movement rates would vary among translocated animals based on sex, as well as the abundance of conspecifics in the source habitat versus destination habitat(s). Specifically, we predicted greater home ranges and space use of males compared to females, of raccoons originating from low- versus high-density habitats, and of individuals moved to high-density versus low-density habitats.

## Results

Our dataset for statistical analysis included 20 control, 17 transient, and 15 resident states from 26 individual raccoons (Table 1). The four animals that were excluded included 1 that was immediately translocated upon first capture but could not be relocated (no control or translocation data), and three that produced location data too sparse to thoroughly analyze (< 5 locations per week). Two animals dropped the collar during their transient phase. However, we included the data until the date of collar loss (durations of 83 and 42 d) because it was informative for examining transient space use, which resulted in an unequal number of transient and resident states in our analysis. The average duration of the 15 complete transient periods was 36.5 ± 30.0 d, range = 3.3–92.8.

**Table 1:** Sample size (number of individuals) of male and female raccoons translocated between bottomland hardwood and upland pine habitats on the Savannah river site, Aiken SC (2018–2019).

Treatment	Movement phases	Female	Male	Total
Bottomland hardwood to upland pine/riparian	Pre- and post-translocation	5	4	9
	Post-translocation	1	2	3
Upland pine to upland pine	Pre- and post-translocation	0	1	1
	Post-translocation	3	1	4
Upland pine to bottomland hardwood	Pre- and post-translocation	3	3	6
Bottomland hardwood control		1	1	2
Upland pine control		3	2	5
Total		16	14	30

In addition to the 15 complete transient periods used in statistical analysis, we used three of the translocated individuals with sparse data in our qualitative analysis because we were able to determine their final location. Behavior of translocated raccoons could broadly be categorized in three ways: (1) remaining in the vicinity of the release site (final location < 3 km from release location and > 16 km from capture, n = 5); (2) movement from release point to a new home range (final location > 5 km from release location and > 13 km from capture, n = 10; Fig. S2); (3) movement from release point back to the capture location (final location < 4 km from capture, n = 3). One of the animals from the first category never moved more than 2 km from its release location (Fig. S3), whereas the others traveled more broadly before returning to the release location where they remained for the duration of monitoring (Fig. S4).

One of the males that established a new home range away from the release point was incidentally recaptured on 29-Oct 2021 as part of ongoing trapping efforts. It was originally collared on 19-Sept 2018 and translocated on 3-Dec 2018. The animal was recaptured 16.2 km from its original capture location, 9.2 km from its release location, and 1.4 km from its final location recorded on 28-Apr 2019. Of raccoons that returned to the pre-translocation capture location, two had a final location less than 700 m from the capture location, which took them 81 and 54 days to reach, respectively. The other individual from this category was a male with final location 3.8 km from capture location (Fig. S5). This animal was immediately translocated and thus we lacked the data to precisely determine its home range prior to translocation. However, given that the mean 95% UD of males in the control phase was 2.68 km^2^ (range 0.35–7.74) it is reasonable to assume this animal had navigated back to its pre-translocation home range. Interestingly, this animal had among the shortest transient periods of all animals, travelling over 18 km from the release point to this final location in less than 4 days.

Four animals (2 males, 2 females) crossed the Savannah River to Georgia after translocation before crossing back to South Carolina (Fig. S6). Two of these were animals that navigated back to their pre-translocation capture locations. No animals from the high-density source habitat, all of which had access to the river based on their home range locations, crossed it during the control period (a cumulative total of 326 nights of monitoring). Time spent on the Georgia side of the river included 7 days (n = 2), 4 days (n = 1), and less than 24 h (n = 1). Distances between crossing locations (i.e. distance from where they initially crossed from SC to GA and then subsequently crossed from GA back to SC) for each raccoon were 0.5 km, 4 km, 11 km, and 14 km.

**Figure 1:**
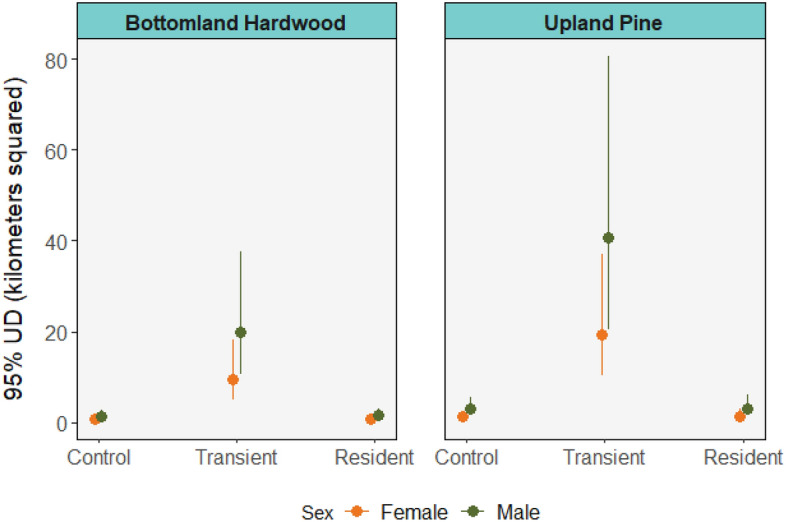
Mean 95% utilization distribution areas with standard error of translocated raccoons monitored on the Savannah River Site during control (pre-translocation), transient (exploratory post-translocation) and resident (stable post-translocation) phases separated by source habitat.

Estimated 95% UDs of translocated animals were approximately 13 times greater than those of control or resident animals (p < 0.001 for both cases), but there was no difference in 95% UD area between the control and resident animals (p = 0.994; top model *w*_i_ = 0.27, multiple *R*^2^ = 0.618; Table 2, Table S1). The estimated 95% UDs of males across all phases were 108% greater than those of females, and animals from low-density habitats had 95% UDs that were 106% greater than those from high-density habitats across all phases (Fig. 1; Table 1).

**Table 2:** Mean and standard deviation (SD) of 95% and 60% utilization distributions and mean movement metrics of translocated raccoons monitored on the Savannah River Site (2018–2019).

Sex	State	Source habitat	*n*	Mean 95% UD (km^2^)	95% UD SD (km^2^)	Mean 60% UD (km^2^)	60% UD SD (km^2^)	Mean hourly distance moved (km/h)	Mean nightly displacement (m)	Mean distance between dens (km)
Female	Control	Bottomland hardwood	5	1.48	1.27	0.26	0.12	0.221	377.59	0.419
		Upland pine	6	1.29	0.43	0.27	0.18	0.180	673.57	0.386
	Transient	Bottomland hardwood	3	11.20	12.30	1.03	1.06	0.244	593.67	0.522
		Upland pine	4	43.70	15.50	4.86	2.70	0.515	2143.80	2.54
	Resident	Bottomland hardwood	4	0.61	0.61	0.14	0.16	0.151	158.51	0.114
		Upland pine	5	1.58	0.87	0.39	0.23	0.234	394.65	0.430
Male	Control	Bottomland hardwood	4	2.53	3.50	0.56	0.71	0.284	402.66	0.204
		Upland pine	5	2.81	0.80	0.69	0.22	0.350	571.22	0.628
	Transient	Bottomland hardwood	5	49.10	61.70	3.68	3.89	0.439	1200.60	0.772
		Upland pine	3	33.00	17.30	5.30	3.32	0.765	2896.1	2.850
	Resident	Bottomland hardwood	5	3.19	1.67	0.71	0.39	0.332	504.92	0.521
		Upland pine	3	3.35	1.64	0.46	0.16	0.240	306.10	0.313

States are control (pre-translocation), transient (exploratory post-translocation) and resident (stable post-translocation).

**Figure 2:**
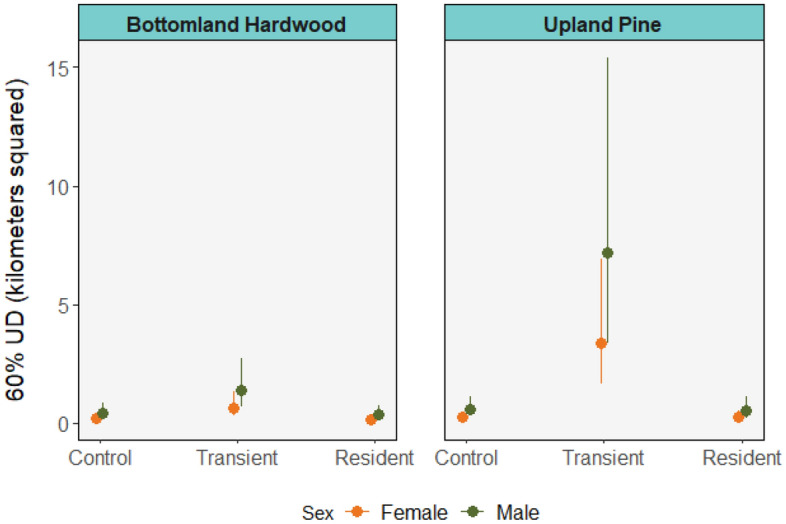
Mean 60% utilization distribution areas with standard error of translocated raccoons monitored on the Savannah River Site during control (pre-translocation), transient (exploratory post-translocation) and resident (stable post-translocation) phases separated by source habitat.

Although the 60% UDs were smaller than the 95% UDs (overall means 1.54 ± 2.36 km^2^ vs 13.43 ± 25.98 km^2^, respectively), the relative effect of sex was similar, with predicted 60% UDs of males to be 106% greater than those of females (top model *w*_i_ = 0.19, multiple *R*^2^ = 0.566; Table S2). There was no difference in 60% UDs between animals from high- and low-density source habitats in either the control (*p* = 0.472) or resident state (*p* = 0.484), but during the transient state 60% UDs were 5.2 times greater for animals from low- compared to high-density habitats (p = 0.001; Fig. 2).

**Figure 3:**
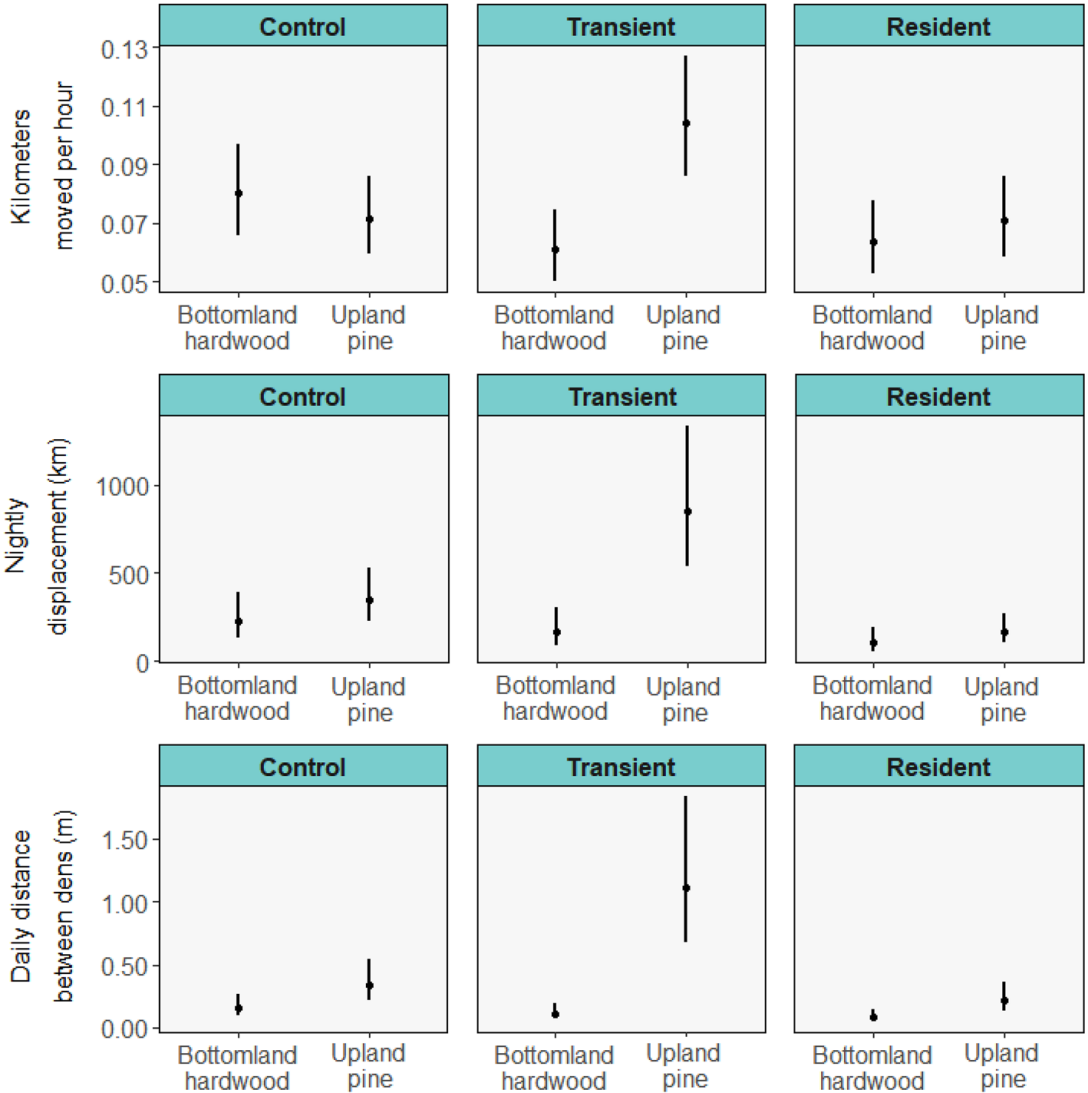
Mean values with standard errors of movement metrics of translocated raccoons monitored on the Savannah River Site (2018–2019) during control (pre-translocation), transient (exploratory post-translocation) and resident (stable post-translocation) phases separated by source habitat.

**Figure 4:**
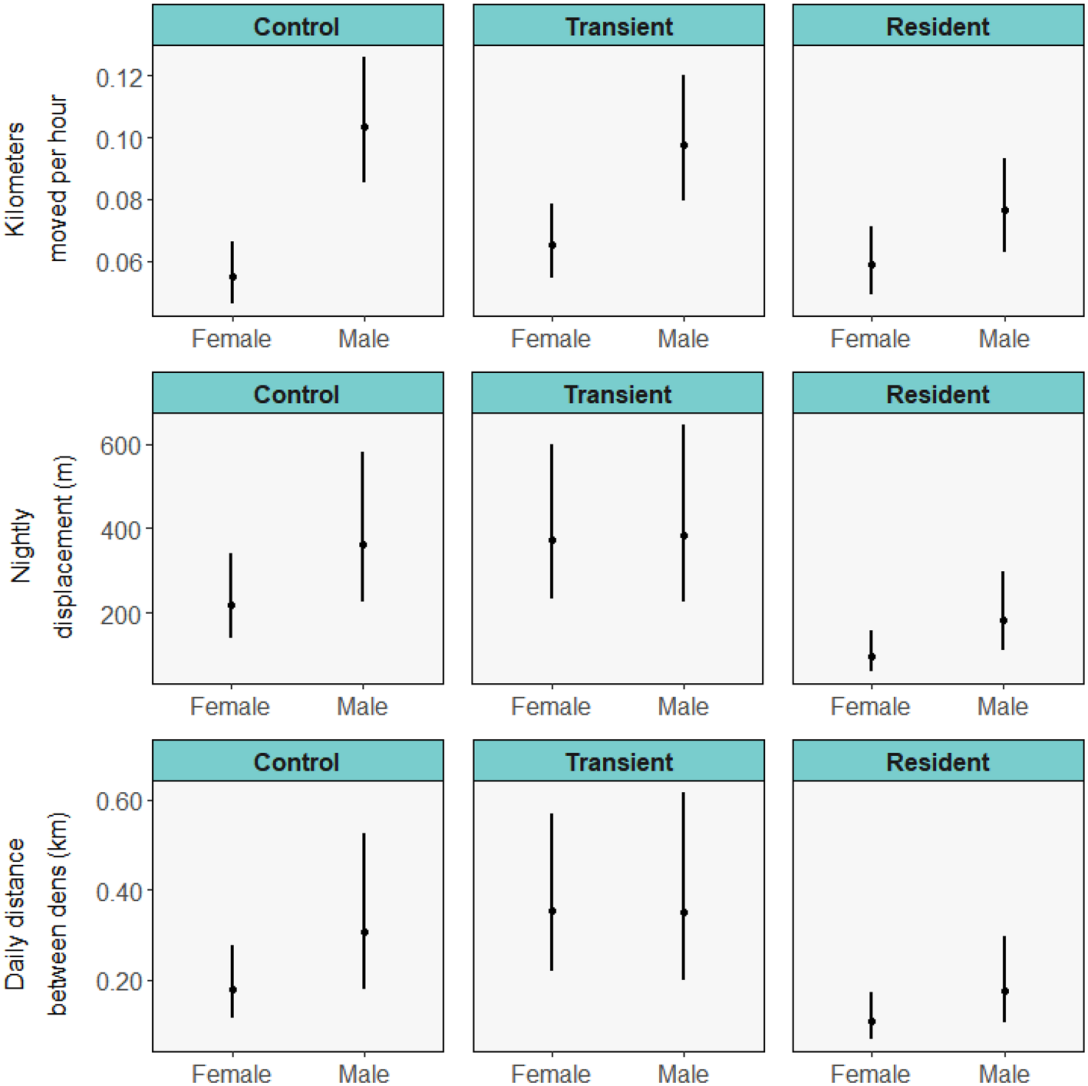
Mean values with standard errors of movement metrics of translocated raccoons monitored on the Savannah River Site (2018–2019) during control (pre-translocation), transient (exploratory post-translocation) and resident (stable post-translocation) phases separated by sex.

There was no difference in kilometers moved per hour between high- and low-density source habitats for control (*p* = 0.759) or resident states (*p* = 0.182), but animals from low-density source habitats moved twice the distances per hour of those from high-density habitats during the transient state (*p* < 0.001; Fig. 3; *w*_*i*_ = 0.21, marginal *R*^2^ = 0.031, conditional *R*^2^ = 0.093; Fig. 3; Table S3). Distances moved per hour were similar between resident males and females (*p* = 0.159), but males moved greater distances per hour than females in control (*p* < 0.001) and transient (*p* = 0.022) states (Fig. 4).

Nightly displacement was 5.04 times greater for animals from low-density compared to high-density source habitats during the transient phase (*p* < 0.001; top model *w*_*i*_ = 0.22, marginal *R*^2^ = 0.145, conditional *R*^2^ = 0.292; Fig. 3; Table S4), but not different during the control or resident phase. Animals translocated to low-density destination habitats had a nightly displacement 3.08 times that of those moved to high-density habitats during the transient phase (*p* = 0.0103). Males and females had similar nightly displacement during the control and transient phase, but males moved 1.87 times more than females during the resident phase (*p* = 0.049; Fig. 4).

Compared to animals moved to high-density habitats, animals moved to low-density habitats had twice the average distance between dens (top model *w*_*i*_ = 0.14, marginal *R*^2^ = 0.159, conditional *R*^2^ = 0.256; Table S5). Distance between dens was higher for animals originating from low-density habitats compared to high-density habitats in every state, but the difference was greater for animals during the transient phase (10.5 times larger) compared to the other states (control and resident 2.30 and 2.85 times greater, respectively; Fig. 3). Males had greater distance than females between dens during the control state (*p* = 0.05), but not during the other states (Fig. 4).

## Discussion

Wildlife translocation can be a significant contributor to the spread of zoonotic diseases, and the movement behavior of translocated animals influences the nature and frequency of spatial contacts for disease transmission^2,39^. We found that translocation influenced raccoon movement behavior in ways that may increase social contacts among conspecifics, as it led to an estimated 13-fold increase in home range sizes. These home range expansions may correspond to greater overlap with conspecifics and an increase in contacts with unique individuals^40^. In addition to potentially introducing rabies or other diseases into a new region, the increased movement rates of translocated individuals result in a high risk of spreading the disease to other animals once introduced.

Most raccoons adhered to the paradigm and general patterns observed for translocated wildlife, characterized by immediate departure from the release point followed by large increases in space use compared to non-translocated individuals^26,41,42^. This behavior accounts for the negligible influence of destination habitat on post-translocation behavior because most animals did not remain close to their release location. Extensive movement may help translocated animals orient to find shelter or other resources in a novel environment^24^. Additionally, wide-ranging movements may reflect attempts to navigate back to their original home range areas^24,41^. Indeed, three animals traveled back to their capture location, indicating the possibility of homing by raccoons across these spatial scales. Rosatte and MacInnes^43^ experimentally moved 24 raccoons distances of 25–45 km from urban Toronto into surrounding rural areas, but did not document homing, leading them to conclude that raccoons did not possess homing capabilities. Our study results on a slightly smaller spatial scale contradict this notion, with one animal immediately moving back toward its capture location, arriving back to its original home range within 4 days.

The large home ranges post-translocation suggest that many animals attempted to return to their pre-translocation home ranges, but it is unclear why only 16% of animals were successful. For some carnivores, males are more likely to home successfully^41^, but we were unable to firmly conclude this based on our sample size, and one of the individuals that homed was a female. There is also typically a translocation distance threshold beyond which animals have a diminished homing ability^42^. Considering the lack of homing documented by Rosatte and MacInnes^43^ at distances of 25–45 km, the distances of 16–20 km that we translocated raccoons may represent the upper bounds of distances over which raccoons are capable of homing. Alternatively, there may not have been ample environmental cues for raccoons to locate their pre-translocation home ranges. Translocated wild boar, for example, were able to home when they located portions of streams that were within their original home ranges^26^, and raccoons may have been more successful at homing had there been more prevalent environmental cues for navigation.

There is substantial intraspecific variability in animal behavior which may be further amplified by translocation. For some animals, the lack of homing occurred because they never tried to move back to the capture location, instead remaining in the vicinity of the release point. Variation in behavioral responses to translocation was observed despite all animals being moved similar distances. The duration of the transient phase varied extensively, but was not related to homing tendencies as animals that homed represented both the longest and shortest transient durations. Likewise, translocated tigers (*Panthera tigris*)^44^, fishers (*Pekania pennanti*)^45^, and American martens (*Martes americana)*^46^ varied widely in their post-translocation home ranges and time to establish residency. Despite variation during the transient period, similarities in home range sizes between the control and resident periods indicate that following the transient period, raccoons generally resume typical movement patterns, similar to the findings of Rosatte and MacInnes^43^. It is possible that further changes in movement behavior or home range sizes may have occurred following the period of monitoring. However, the incidental trapping of a translocated animal in our study less than 1.5 km from its last known location 2.5 years later suggests that raccoons may permanently settle in the home ranges they establish post translocation.

Several translocated animals crossed the Savannah River, indicating that translocation alters how some animals use the landscape. Rivers are a barrier that may slow the spread of rabies where they constrain raccoon movements^47–49^. Indeed, raccoons never crossed the Savannah River during the 326 cumulative nights of control phase monitoring, even though all high-density source habitat (i.e. bottomland hardwood) was located within a few kilometers of the river. One of the translocated raccoons originally captured in the high-density habitat crossed the river during the translocation period before moving back to its original home range. After returning to its original location, it did not cross the river again. Similarly, across barrier islands in Virginia, USA, translocated raccoons frequently traversed open water and expansive marshes to move back to their origin, whereas non-translocated animals rarely made overwater movements^27^. Translocation thus appears to result in atypical movement behaviors such as crossing physiographic barriers as animals seek to establish a new home range or find their habitat of origin. In addition to increases in space use, the breaching of natural barriers to disease spread further increases the risk that translocation will contribute to rabies or other disease transmission.

Following our predictions, male raccoons had larger home ranges compared to females across the entire period of observation likely due to greater resource requirements and differences in mating habits^31–33^. These divergences in space use in terms of UD sizes were generally present in equal magnitude after translocation, suggesting that translocations did not affect sex-specific patterns in space use. Fishers^45^ and tigers^50^ also maintained sex-specific patterns in space use following translocation. Dispersal distance of translocated mammals is proportional to home range size^51^, with greater home ranges of males likely contributing to their greater space use during the transient period. Thus, the underlying ecological factors contributing to differences in home range sizes between the sexes appear to be unaffected by translocation. The greater space use of males compared to females during the transient phase could result in males having greater potential to transmit disease upon translocation.

The source habitat was consistently an influential factor impacting space use following translocation. In accordance with our predictions, individuals originating from low-density habitats exhibited greater movement compared to those from high-density habitats, which also occurred for wild boar translocated on the SRS^26^. As a result of living in closer proximity to conspecifics, animals from high-density habitats likely have experienced increased rates of conspecific encounters prior to translocation. As such, they may be less likely to avoid other raccoons in the destination habitat, directly competing with them for resources and exhibiting smaller home ranges as a result. By contrast, animals from low-density habitats may be more apt to avoid conspecifics due to their history of less frequent interspecific interaction, leading them to exhibit wider ranging behavior as they seek to avoid other raccoons. Alternatively, patterns in space use may carry over following translocation based on prior experience^34,35^. In low-density habitats, raccoons likely have larger home ranges due to less spatial concentration of resources, whereas the opposite is true for high-density habitats^36^. The patterns in movement following translocation may therefore be a vestige of habitat-specific movement patterns in their native habitats.

Raccoon densities across our high- and low-density habitats are relatively low compared to other land cover types such as urban or agricultural^15,52,53^. Considering the influential role of source habitat on post-translocation movement behavior, raccoons from these higher density habitats might have displayed divergent patterns in space use than what we recorded. Destination habitat may have also been influential if we had translocated raccoons to agricultural and urban habitats, as the abundance of resources may have promoted fidelity to the release location^15,52,54^. However, there was not a strong indication that greater resource abundance in the higher quality destination habitat in our study (bottomland hardwoods) influenced movement behavior or site fidelity.

In addition to these considerations, raccoons exhibit seasonal changes in home range size^31,33,55^ and since most of our tracking was carried out in the fall and winter, animals may have shown different patterns in space use during spring and summer. Raccoons have complex social structures^56^ which could impact their movement behavior, but we were unable to account for this in our study. Lastly, our marginal R^2^ values for many of the movement metrics were relatively low, indicating that our models only explained a small amount of variation in these variables. Individual variability among animals tended to account for a greater share of variability in our models than fixed effects as the conditional R^2^ values were typically about twice as large as the marginal R^2^ values. However, our models for UD sizes had much larger R^2^ values, indicating greater ability of these models to explain variation in home range sizes.

Translocation has been a commonly used technique to manage human-wildlife conflict, but its utility remains widely debated^41,57^. Adverse consequences of raccoon translocation include increased mortality and the continuation of conflict-causing behavior in the destination habitat^24,43^. The homing behavior that we documented may also temper the effectiveness of translocation because animals can potentially return to their capture location. Raccoon translocations have been most commonly associated with the spread of rabies, but raccoons are also vectors for other diseases including bovine tuberculosis^58^, Chagas disease^59^, canine distemper^60^, leptospirosis^61^, raccoon roundworm^62^ and visceral larva migrans^63^. Large increases in space use combined with the breaching of natural barriers results in considerable risk of disease spread by raccoons following translocation. These findings underscore the need for alternative management strategies to safeguard current rabies control efforts and prevent further spread of wildlife rabies viruses in eastern North America.

## Methods

### Study area

**Figure 5:**
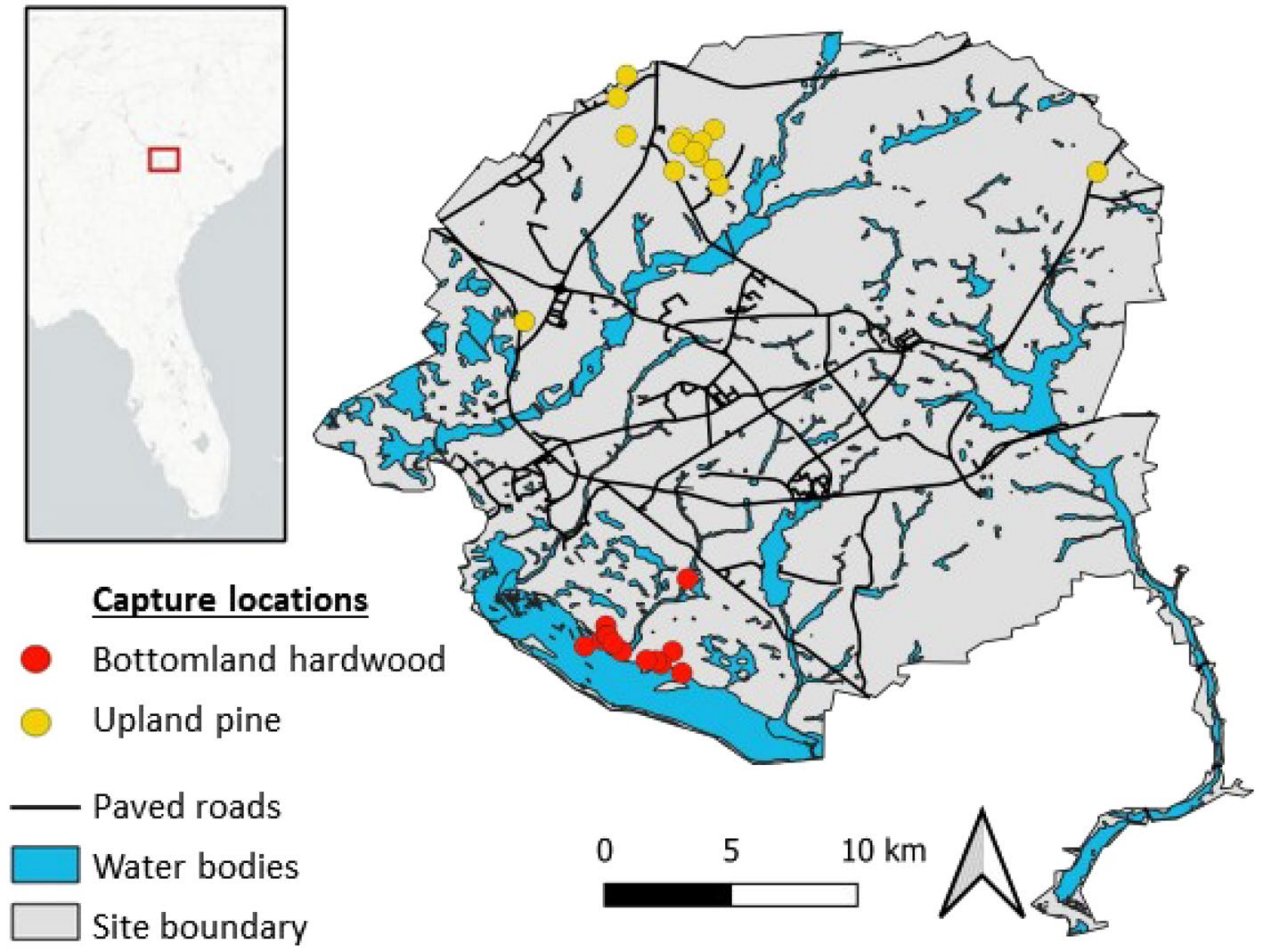
Map of the Savannah River Site with capture locations of raccoons from bottomland hardwood (red) and upland pine (yellow) habitats. Figure created using QGIS version 3.22 (https://www.qgis.org/en/site/).

We conducted this study at the Savannah River Site (SRS), a property owned by the US Department of Energy that encompasses 78,000 ha in the upper coastal plain region of South Carolina across Aiken, Allendale, and Barnwell counties (33°19′N, 81°42′W; Fig. 5). The southwestern border of the site is formed by the Savannah River, which is slow-moving and approximately 100 m wide in this region, with a floodplain that extends to 3 km^64^. Dredging operations have maintained a 2.7-m deep navigation channel^65^. SRS is mostly covered by evergreen forest (54%) and woody wetlands (24%), with other land cover types (e.g. developed, open water, mixed forest) collectively comprising 22% of the land area^66^. Much of SRS has been managed for timber harvest since 1951 and stands are harvested on a rotating basis. Predominate forest types are loblolly pine (*Pinus taeda*), longleaf pine (*Pinus palustris*), and bottomland hardwoods (e.g. *Nyssa* spp., *Quercus* spp.)^67^. Raccoons are present in habitats across the site^68^.

### Experimental design

We used a replicated full-factorial study design which consisted of reciprocal translocations of raccoons between high-density (bottomland hardwood/riparian) and low-density (upland pine) raccoon habitats. We completed four relocation experiments with raccoons based upon expected differences in conspecific abundance comparing source and destination habitats: high- to low-density, low- to high-density, high- to high-density, and low- to low-density. All translocations were conducted at a minimum of 16 km between source and destination habitats.

We chose high- and low-density habitat types based on predicted raccoon densities resulting from habitat-specific resource availability^33,69,70^, as we did not have habitat-specific raccoon abundance estimates across the site when the experiment was conducted. Bottomland hardwoods were classified as high-density because they tend to be more preferred by raccoons compared to upland pine forests due to the presence of suitable denning trees, free water, and foraging opportunities^17,33,69,70^. Bottomland hardwoods are confined to the lower southwest portion of the site along the Savannah River and consist of seasonally flooded cypress-tupelo forests (*Taxodium distichum*-*Nyssa aquatica*), with oak (*Quercus* spp.) and hickory (*Carya* spp.) scattered throughout^67^.

Upland pine was considered a low-density habitat because they generally have less abundant resources for raccoons^55,70^. During the fall and winter when this study was carried out, food resources are especially sparse for raccoons due to the absence of soft mast^55,70^. Free water in upland pine on our site is also less prevalent than in bottomland hardwoods. Additionally, large diameter denning tress are less common than in bottomland hardwoods because most stands are managed for timber harvest. Pine stands are subject to prescribed burning at SRS, which may further reduce habitat resources for raccoons^33,67^.

Riparian habitats were used only as the destination for raccoons moved from high- to high-density habitats. We chose riparian habitats in these instances because we were unable to maintain a minimum distance of 16 km between capture and release locations for an animal moved between bottomland hardwood locations due to their spatial constraint on the site. We chose riparian sites because they have similar tree species composition as bottomland hardwoods and are in proximity to water, so we assumed similar resources and resulting raccoon densities^69,71^.

A mark-recapture study to estimate habitat-specific raccoon densities was conducted concurrently with this work and was used to test our assumptions regarding habitat-specific densities^68^. Our assumptions regarding habitat-specific densities were not fully supported, as there was no difference in raccoon densities between riparian (the high-density destination habitat; estimated density = 2.19 ± 0.29 animals/km^2^) and upland pine (the low-density source and destination habitat; estimated density = 2.14 ± 0.23 animals/km^2^) habitats^68^. However, our assumptions regarding bottomland hardwood densities were supported, as raccoon densities were significantly higher than in upland pine (bottomland hardwood estimated density = 5.44 ± 0.37 animals/km^2^). Thus, there were no animals moved from high-to high-density habitats as planned and animals that were assigned the high- to high-density treatment were in reality moved from high- to low-density habitats. Consequently, we considered animals moved from bottomland to riparian as being moved from high- to low-density.

### Animal capture and handling

We captured animals between September 2018 and February 2019 by setting up transects along secondary SRS roads at sites in bottomland hardwood and upland pine habitats. We placed Tomahawk® model 108SS live-capture box traps (Hazelhurst, WI) along transects100 meters apart from one another and covered them with brush. Traps were baited with corn and plaster tabs soaked in fish oil^72^ and rebaited every 5 days.

Upon capture, raccoons were immobilized using intramuscular injection of Telazol (Fort Dodge Animal Health, Fort Dodge, IA) at a dosage of 5 mg/kg of estimated body weight^73–75^. Once anesthetized, we determined sex, age, and body mass and gave each raccoon a unique numbered ear tag (Monel #3, National Band and Tag Company, Newport, KY). To ensure the collar weight (115 g) was less than 5% of total animal weight, only adult animals that weighed 2.3 kg or more received transmitters^76,77^. We collected spatial data using GPS telemetry data logging transmitters (W500-AA, Advanced Telemetry Systems, Isanti, MN) programed to collect data points once every two hours between 18:00 and 6:00 h, plus an additional point at 12:00. We chose this sampling schedule to maximize battery life; consistent daytime locations were of limited use because raccoons are mostly nocturnal and typically inactive in dens during the day. Each collar was outfitted with a very high frequency (VHF) transmitter that allowed raccoons to be located via radio telemetry for recapture and collar downloads.

Following capture and processing, all raccoons that were not immediately translocated (see below) were released in the same area to collect at least 1 month of pre-translocation movement data. Once the pre-translocation period was complete, we attempted to recapture all collared raccoons by identifying the den locations using radio telemetry and deploying multiple box traps around the den. Once recaptured, we kept the animal in the trap, covered the trap with a blanket to minimize stress, and transported it by vehicle to its assigned destination habitat where the animal was released.

We initially deployed collars on 22 raccoons divided evenly among the sexes during September–October 2018. Animals that could not be recaptured for translocation were considered control animals and used for pre-translocation data. To account for our inability to recapture some of the animals and obtain translocation data, we deployed an additional 8 collars during January–February 2019 in treatments that were lacking translocation data and translocated these animals immediately. As a result, animals provided movement data from one of three possible scenarios: (1) tracked for ≥ 1 month and translocated (both pre-and post-translocation data); (2) not translocated (pre-translocation data only); or (3) immediately translocated upon initial capture (post-translocation data only; Table 1; Fig. S1).

Data were remotely downloaded from each collar using an ultra-high frequency (UHF) antenna during daylight hours while raccoons were denning. We downloaded data at approximately monthly intervals until collar failure, loss of signal, or through July 2019. All animal handling practices conformed to the American Society of Mammologists guidelines^77^. All trapping and handling procedures complied with the ARRIVE guidelines and were performed in accordance with the relevant guidelines and regulations. The experimental protocols were approved by the Institutional Animal Care and Use Committee of the University of Georgia (Animal Care and Use Protocol A2018 06-024-A12).

### Translocation analyses

To characterize movement and examine individual raccoon homing behavior, we determined the last known location of every translocated animal by calculating the centroid of its final 30 GPS coordinates. We then calculated the distance between this centroid and the release location as well as the distance between the centroid and the original capture location. We also plotted all points using QGIS version 3.22^78^ to examine crossing of landscape features such as water bodies.

**Figure 6:**
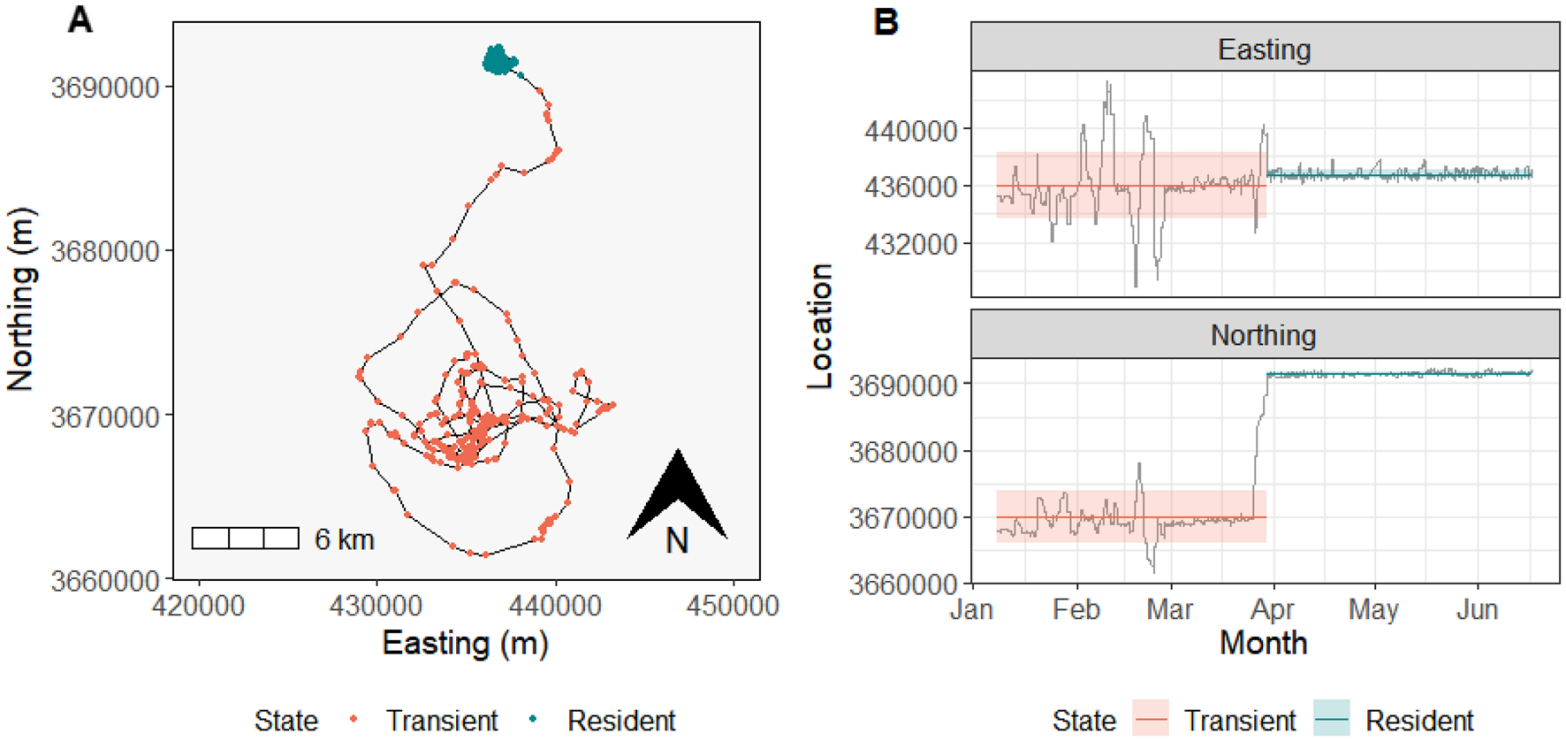
Movements of a raccoon on the Savannah river site translocated on 1/8/2019. Shift from transient to resident state was determined by changes in the mean and variance in coordinate data. Both states lasted ~ 80 days. The map of resident and transient segments is depicted in (**A**) and (**B**) shows the corresponding shifts in location graphically.

We investigated shifts from transient (i.e. exploratory) to resident (i.e. stable) movement behaviors following translocation using the package ‘segclust2d’ in Program R version 4.0.4^79,80^, which identifies shifts in home ranges based on changes in the mean and variance of latitude and longitude through an iterative procedure that combines dynamic programming with expectation–maximization^81^. We defined the home range immediately after translocation as the transient home range. When the transient home range shifted, we defined this new home range as the resident home range (Fig. 6). Thus, the chronology of the 3 types of home ranges examined was: control (pre-translocation) → transient (immediately post-translocation) → resident. We compared characteristics of transient and resident home ranges to control home ranges using data from animals that were never translocated or from the same collared animals during the pre-translocation period. For comparison to controls, we aimed to include control data from approximately the same amount of time over the same period of the year based on the duration of the transient and resident periods. The mean duration of the transient period for translocated animals was 36 days and the mean duration of the residency period was 68 days, and this data spanned from October to March. Therefore, for analysis of control animals, we selected 52 consecutive days at random between the same months.

We examined space use by constructing 95% and 60% utilization distributions (UDs) using dynamic Brownian bridge models for the three home range types separately with the R package ‘move’^82^. We also quantified movement behavior by calculating the nightly displacement (distance between location at 18:00 h and subsequent location at 12:00 h) using the package ‘adehabitatLT’^83^. We also examined the distance moved per hour and the distance between consecutive denning locations. We calculated the latter based on the location at 12:00, assuming animals to be denning at this time.

We compared the log-transformed values of the two UD sizes between the three states (control, transient, resident) using a separate linear model for both UDs. In addition to state, we included sex and conspecific density in both the source and destination habitats as a categorical variable (high or low for both), as well as all possible two-way interactions between the four variables. We compared log-transformed values of the other four movement metrics with a linear mixed effects models using the package ‘lme4’^84^, incorporating individual as a random effect. We included the same four fixed effects and their interactions as in the UD models. We used different models due to differences in the data structure; for the UDs we had a maximum of 3 cases for an individual (a UD from all three movement states) and for some individuals only one (see “results”) which resulted in convergence errors using a mixed effects model. For the other movement metrics, however, there were data for every day of tracking which resulted in much larger sample sizes for every animal and rendered mixed effects models appropriate for analysis.

To evaluate models, we ranked null and all possible model combinations using sample size corrected AIC_c_, selecting the lowest AIC_c_ as the best performing model and making inferences from this top model^85^. We also assessed the relative support for the top model by calculating the Akaike weights (*w*_*i*_) and comparing it to models within two AIC_c_ units (ΔAIC_c_ ≤ 2). If the top model contained state as a variable or an interaction term, we used the package ‘emmeans’ to compute pairwise comparisons using α = 0.05 to determine statistical significance^86^.

## Supplementary Information

Supplementary Information.

## Data Availability

The datasets used during the current study are available from the corresponding author on reasonable request.
