# Supplementary material for: Spatial ecology of translocated raccoons
Source: Sci Rep. 2023 Jun 27;13:10447. doi: 10.1038/s41598-023-37323-6 (PMC10300129; doi:10.1038/s41598-023-37323-6)
Supplement: Supplementary file 1 — Supplementary Information. [file 41598_2023_37323_MOESM1_ESM.docx]

Supporting Information for Hill et al. Spatial ecology of translocated raccoons


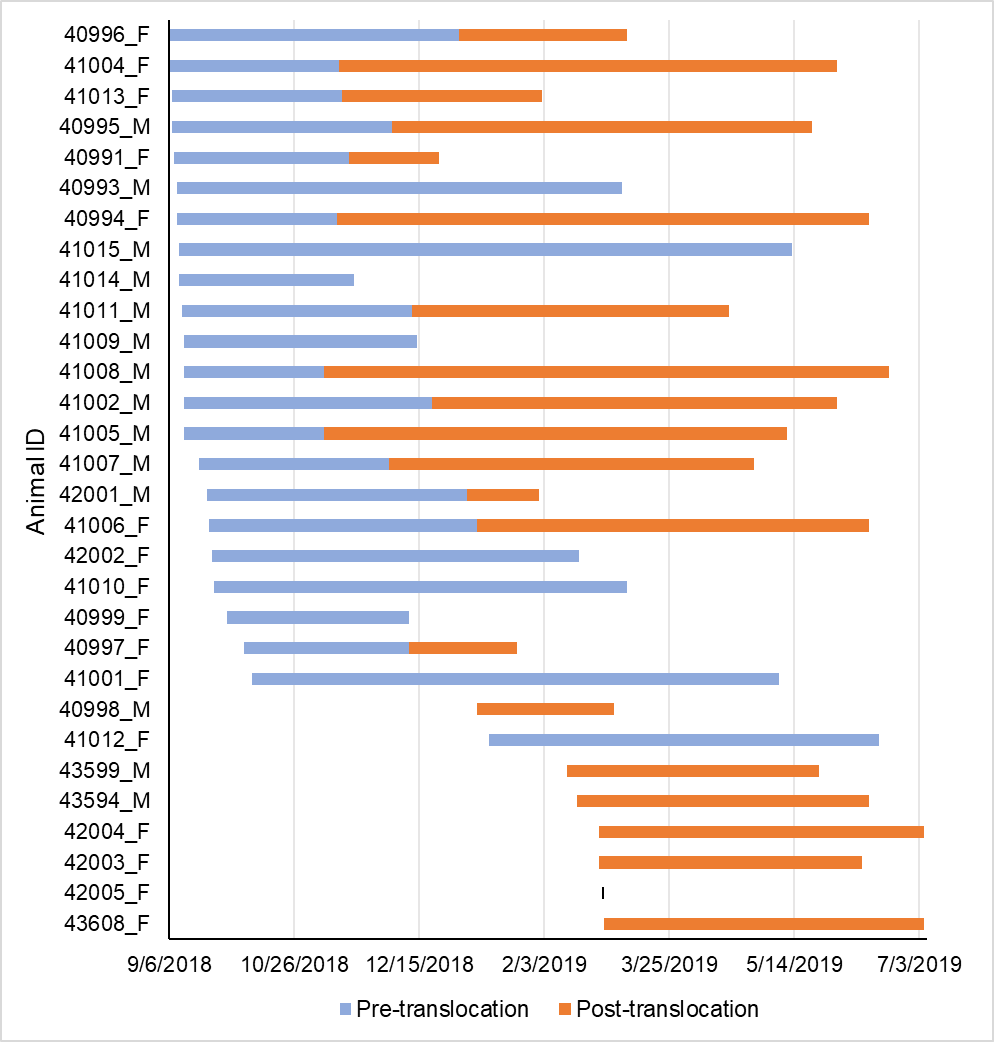


**Figure S1.** Time periods of pre-and post-translocation data collection for 30 raccoons monitored on the Savannah River Site, September 2018-July 2019. Letter after ID indicates male or female. Animals with only pre-translocation data could not be recaptured for translocation or could not be relocated after translocation. Animals with post-translocation data only were immediately translocated following initial capture. Individual 42005 produced no data because it was immediately translocated but could not be relocated.


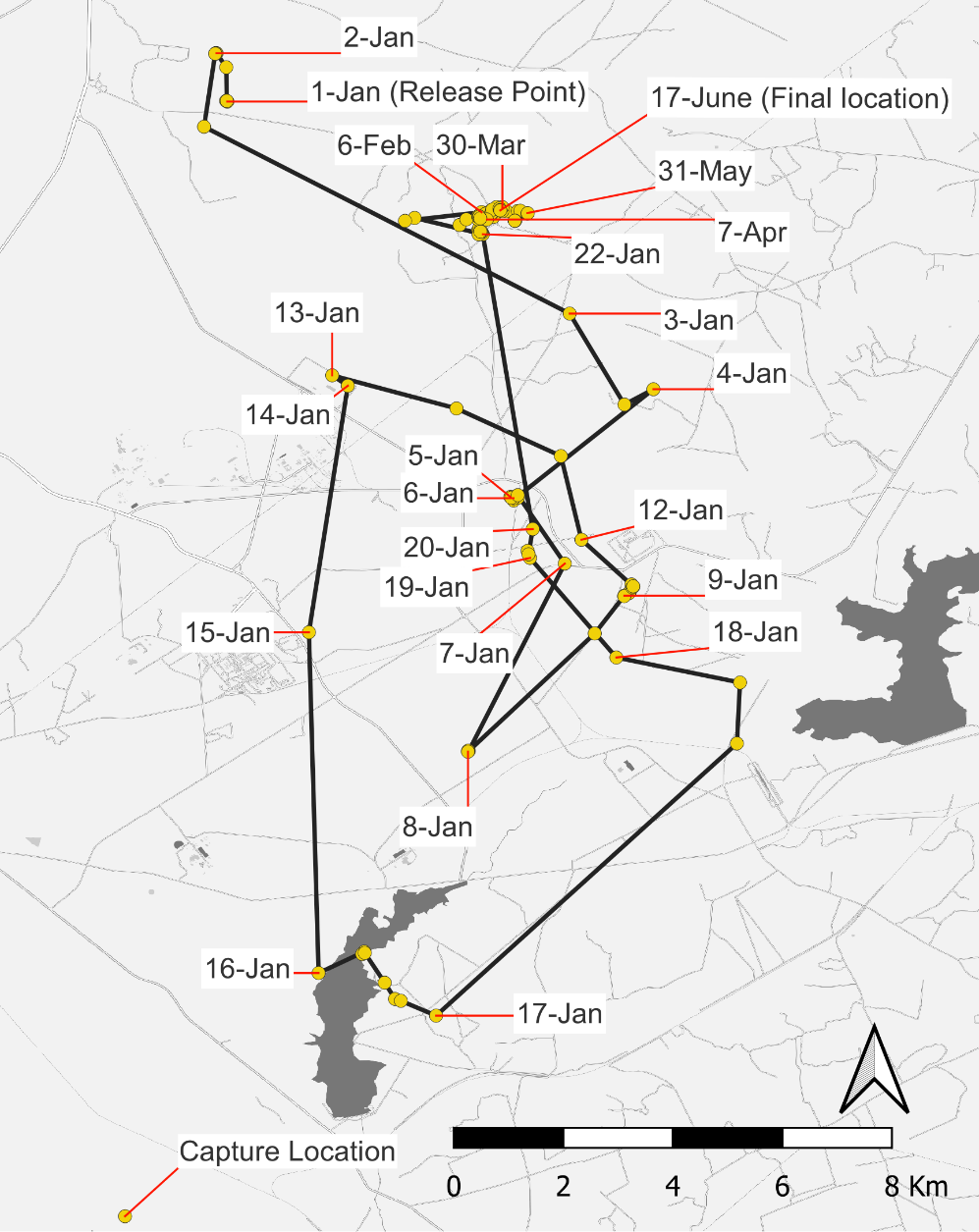


**Figure S2.** Movement from release point to a new home range of a female raccoon (ID 40996) translocated on the Savannah River Site on 1-January 2019. Gray lines represent roads and large dark gray areas indicate water bodies. Figure created using QGIS version 3.22 (<https://www.qgis.org/en/site/>).


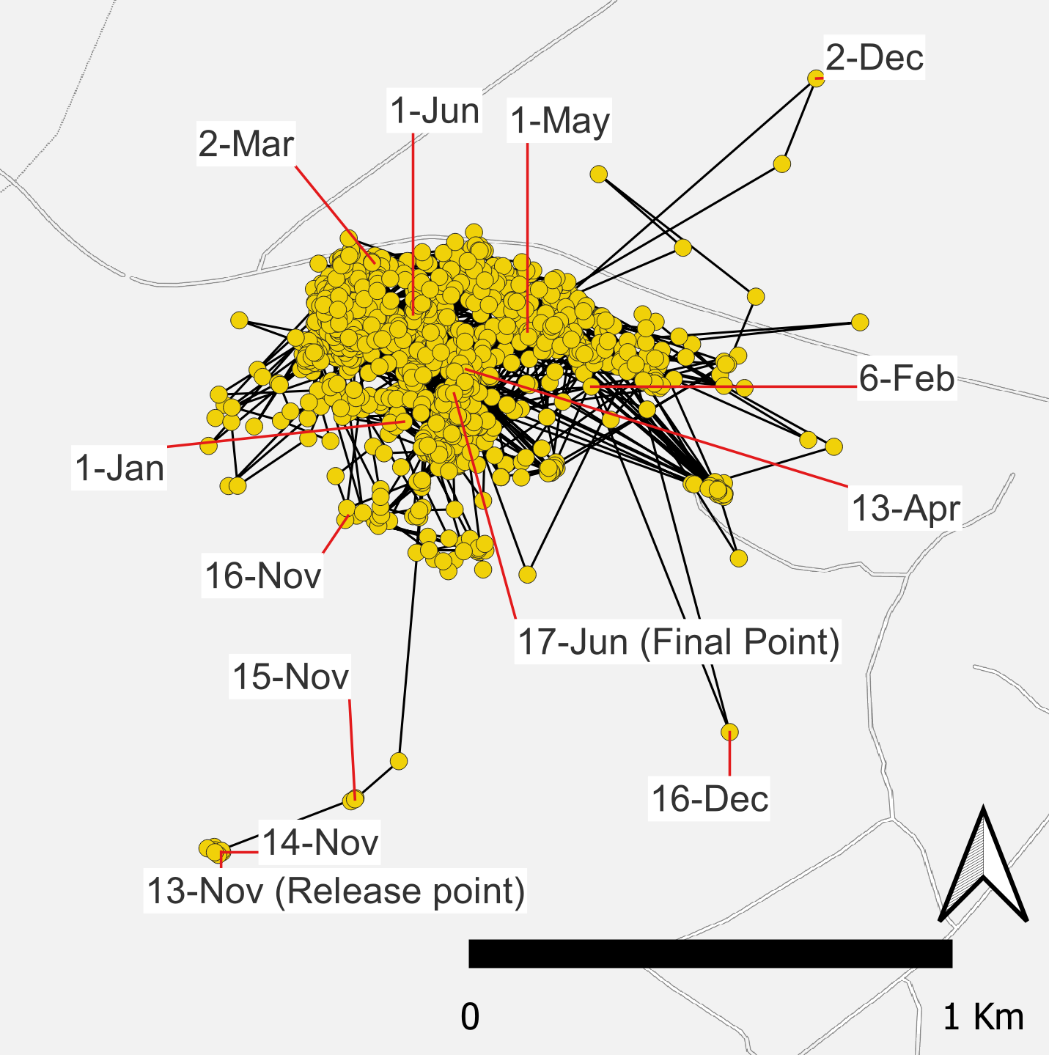


**Figure S3.** Movements of a female raccoon (ID 40994) translocated on the Savannah River Site on 13-November 2018 that remained near the release point. Gray lines represent roads and large dark gray areas indicate water bodies. Figure created using QGIS version 3.22 (<https://www.qgis.org/en/site/>).


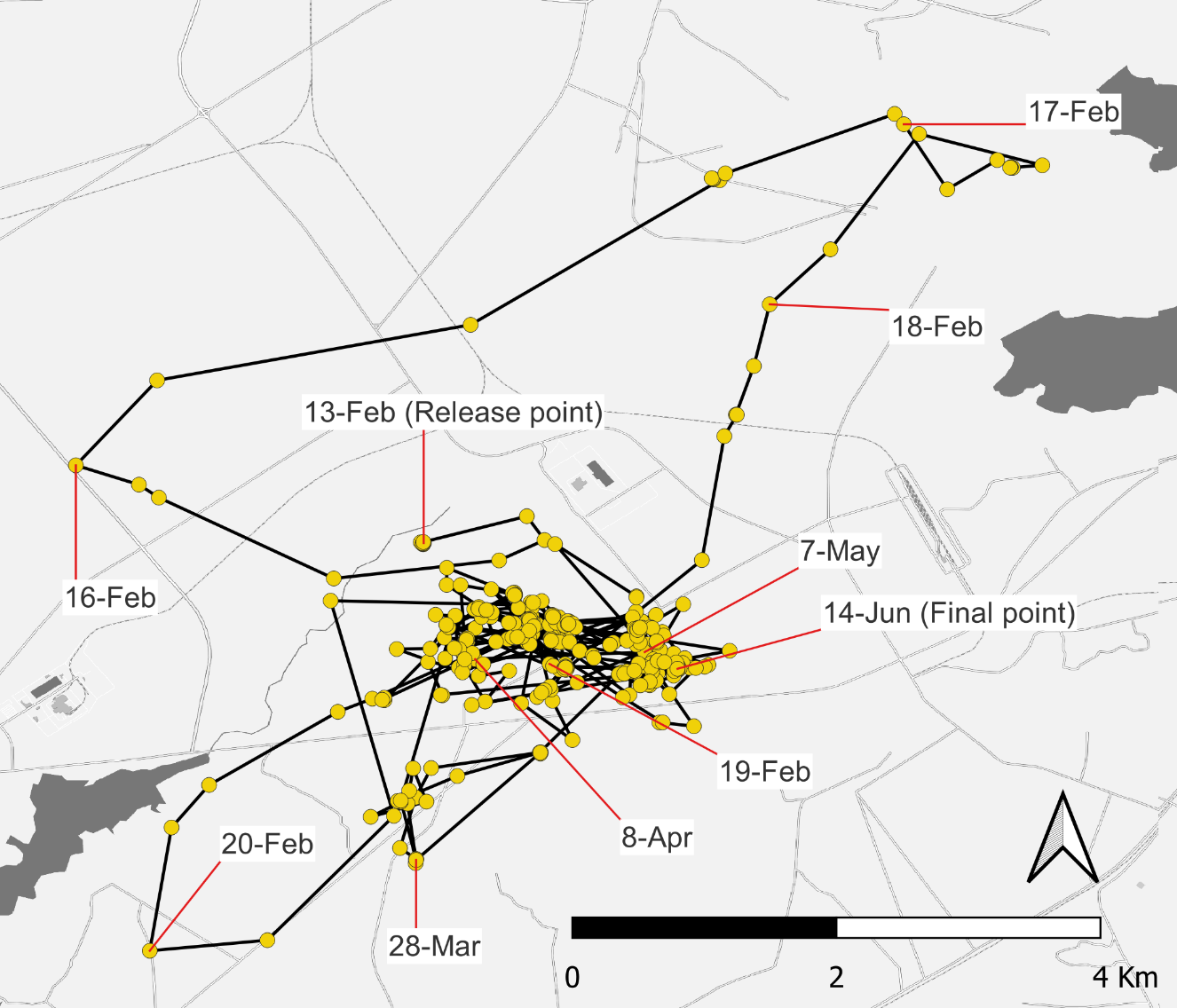


**Figure S4.** Movements away from release point before returning to release point of a male raccoon (ID 43599) translocated on the Savannah River Site on 13-February 2019. Gray lines represent roads and large dark gray areas indicate water bodies. Figure created using QGIS version 3.22 (<https://www.qgis.org/en/site/>).


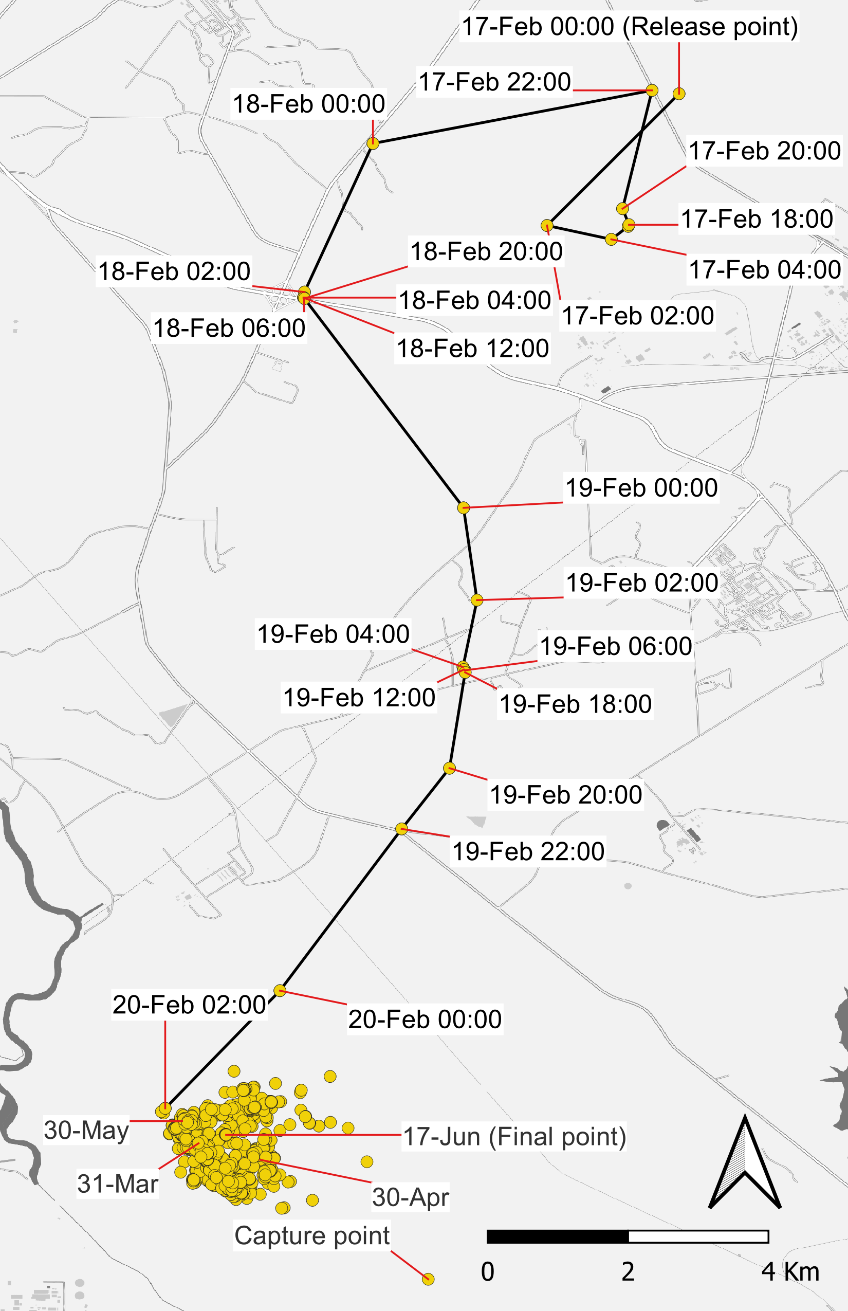


**Figure S5.** Movements back to original capture location of a male raccoon (ID 43594) translocated on the Savannah River Site on 17-Febuary 2019. Gray lines represent roads and large dark gray areas indicate water bodies. Figure created using QGIS version 3.22 (<https://www.qgis.org/en/site/>).


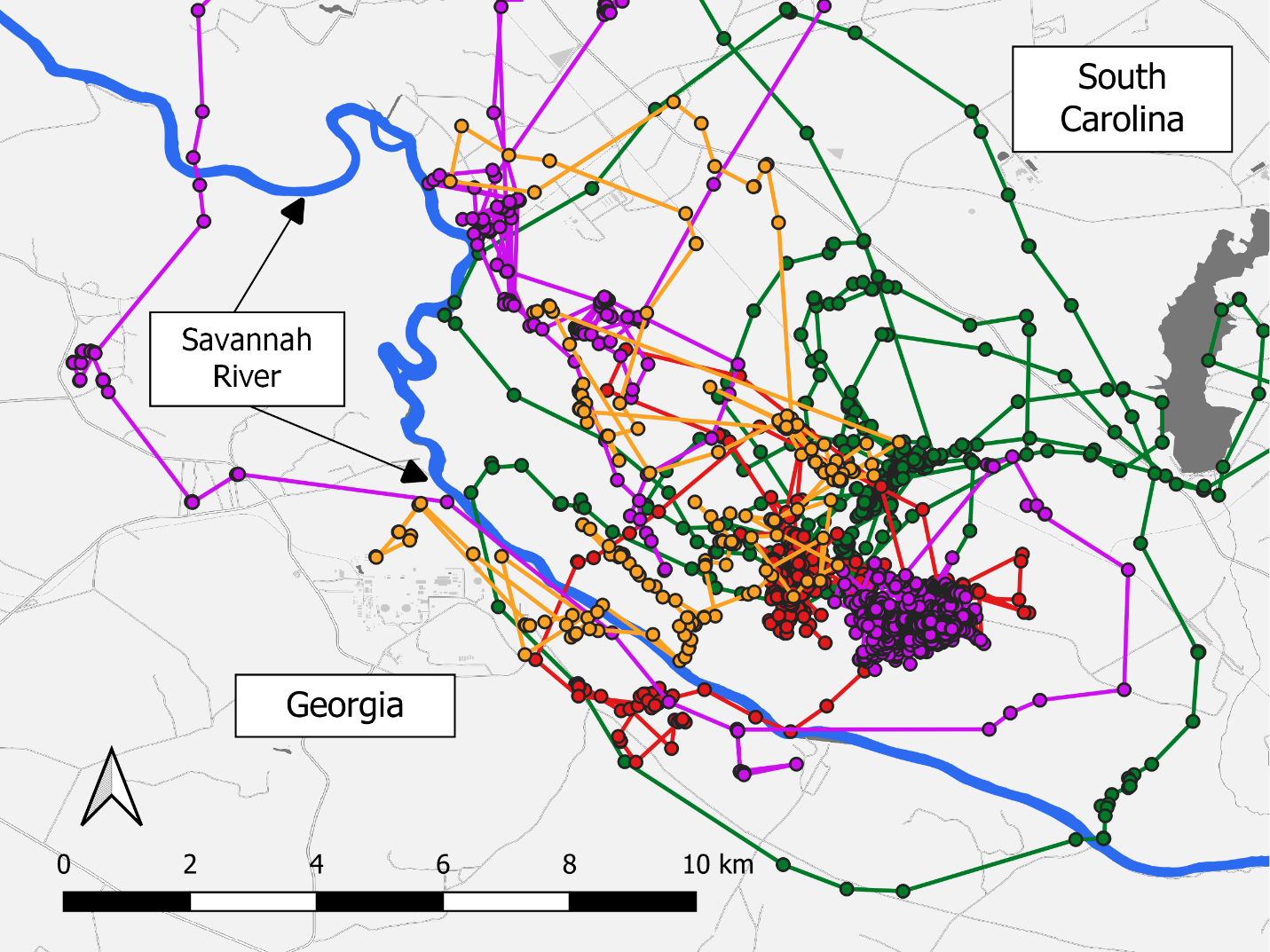


**Figure S6.** Movements of four translocated raccoons showing crossing of the Savannah River between South Carolina and Georgia, USA. Gray lines represent roads and large dark gray areas indicate water bodies, except for the Savannah River, which is shown in blue. Figure created using QGIS version 3.22 (<https://www.qgis.org/en/site/>).

**Table S1.** Linear model comparisons for 95% utilization distribution area of raccoons translocated at the Savannah River Site (2018-2019). Levels of start (habitat where the animal was caught) and end (habitat where animal was moved) and are bottomland hardwood and upland pine. State refers to the translocation status and has 3 levels: control (pre-translocation), transient (exploratory post-translocation) and resident (stable post-translocation). Model output includes sample size corrected Akaike’s information criterion (AIC_c_), Akaike weights (w_i_), log likelihood (LL), number of parameters (*K)*, and difference in AIC_c_ between each model and top model (ΔAIC_c_). Plus sign (+) in columns indicates the parameter was included in the model. Models with 10 lowest AIC_c_ values are presented.

| **End** | **Sex** | **Start** | **State** | **End:Sex** | **End:Start** | **End:State** | **Sex:Start** | **Sex:State** | **Start:State** | ***K*** | ***LL*** | **AIC_c_** | **ΔAIC_c_** | ***w_i_*** |
| --- | --- | --- | --- | --- | --- | --- | --- | --- | --- | --- | --- | --- | --- | --- |
|  | + | + | + |  |  |  |  |  |  | 6 | -74.47 | 162.81 | 0.00 | 0.27 |
|  | + | + | + |  |  |  | + |  |  | 7 | -74.08 | 164.71 | 1.91 | 0.11 |
|  | + | + | + |  |  |  |  |  | + | 8 | -72.97 | 165.29 | 2.48 | 0.08 |
| + | + | + | + |  |  |  |  |  |  | 7 | -74.47 | 165.48 | 2.68 | 0.07 |
|  | + | + | + |  |  |  |  | + |  | 8 | -73.39 | 166.13 | 3.32 | 0.05 |
|  | + |  | + |  |  |  |  |  |  | 5 | -77.45 | 166.20 | 3.40 | 0.05 |
|  |  | + | + |  |  |  |  |  |  | 5 | -77.62 | 166.54 | 3.73 | 0.04 |
|  | + | + | + |  |  |  | + |  | + | 9 | -72.57 | 167.43 | 4.62 | 0.03 |
| + | + | + | + |  |  |  | + |  |  | 8 | -74.08 | 167.51 | 4.70 | 0.03 |

**Table S2.** Linear model comparisons for 60% utilization distribution area of raccoons translocated at the Savannah River Site (2018-2019). Levels of start (habitat where the animal was caught) and end (habitat where animal was moved) and are bottomland hardwood and upland pine. State refers to the translocation status and has 3 levels: control (pre-translocation), transient (exploratory post-translocation) and resident (stable post-translocation). Model output includes sample size corrected Akaike’s information criterion (AIC_c_), Akaike weights (w_i_), log likelihood (LL), number of parameters (*K)*, and difference in AIC_c_ between each model and top model (ΔAIC_c_). Plus sign (+) in columns indicates the parameter was included in the model. Models with 10 lowest AIC_c_ values are presented.

| **End** | **Sex** | **Start** | **State** | **End:Sex** | **End:Start** | **End:State** | **Sex:Start** | **Sex:State** | **Start:State** | ***K*** | ***LL*** | **AIC**_c_ | **ΔAIC**_c_ | ***w****_i_* |
| --- | --- | --- | --- | --- | --- | --- | --- | --- | --- | --- | --- | --- | --- | --- |
|  | + | + | + |  |  |  |  |  | + | 8 | -69.18 | 157.70 | 0.00 | 0.19 |
|  | + | + | + |  |  |  |  |  |  | 6 | -71.93 | 157.73 | 0.03 | 0.19 |
| + | + | + | + |  |  |  |  |  |  | 7 | -71.52 | 159.60 | 1.89 | 0.08 |
| + | + | + | + |  |  |  |  |  | + | 9 | -68.72 | 159.72 | 2.02 | 0.07 |
|  | + | + | + |  |  |  | + |  |  | 7 | -71.70 | 159.94 | 2.24 | 0.06 |
|  | + | + | + |  |  |  | + |  | + | 9 | -68.94 | 160.16 | 2.46 | 0.06 |
|  | + | + | + |  |  |  |  | + |  | 8 | -71.05 | 161.45 | 3.75 | 0.03 |
|  |  | + | + |  |  |  |  |  |  | 5 | -75.26 | 161.82 | 4.12 | 0.02 |
| + | + | + | + |  |  |  | + |  |  | 8 | -71.33 | 162.00 | 4.30 | 0.02 |
| + | + | + | + |  | + |  |  |  |  | 8 | -71.47 | 162.30 | 4.60 | 0.02 |

**Table S3.** Generalized linear model comparisons for hourly distance moved of raccoons translocated at the Savannah River Site (2018-2019). Levels of start (habitat where the animal was caught) and end (habitat where animal was moved) and are bottomland hardwood and upland pine. State refers to the translocation status and has 3 levels: control (pre-translocation), transient (exploratory post-translocation) and resident (stable post-translocation). Individual was included as a random effect. Model output includes sample size corrected Akaike’s information criterion (AIC_c_), Akaike weights (w_i_), log likelihood (LL), number of parameters (*K)*, and difference in AIC_c_ between each model and top model (ΔAIC_c_). Plus sign (+) in columns indicates the parameter was included in the model. Models with 10 lowest AIC_c_ values are presented.

| **End** | **Sex** | **Start** | **State** | **End:Sex** | **End:Start** | **End:State** | **Sex:Start** | **Sex:State** | **Start:State** | ***K*** | ***LL*** | **AIC_c_** | **ΔAIC_c_** | ***w_i_*** |
| --- | --- | --- | --- | --- | --- | --- | --- | --- | --- | --- | --- | --- | --- | --- |
|  | + | + | + |  |  |  |  | + | + | 11 | -22792.91 | 45607.84 | 0.00 | 0.21 |
| + | + | + | + |  |  |  |  | + | + | 12 | -22792.08 | 45608.18 | 0.35 | 0.18 |
| + | + | + | + | + |  |  |  | + | + | 13 | -22791.25 | 45608.52 | 0.69 | 0.15 |
|  | + | + | + |  |  |  | + | + | + | 12 | -22792.91 | 45609.84 | 2.00 | 0.08 |
| + | + | + | + |  | + |  |  | + | + | 13 | -22791.97 | 45609.98 | 2.14 | 0.07 |
| + | + | + | + |  |  |  | + | + | + | 13 | -22792.04 | 45610.11 | 2.27 | 0.07 |
| + | + | + | + | + |  |  | + | + | + | 14 | -22791.21 | 45610.45 | 2.61 | 0.06 |
| + | + | + | + | + | + |  |  | + | + | 14 | -22791.23 | 45610.49 | 2.66 | 0.06 |
| + | + | + | + |  | + |  | + | + | + | 14 | -22791.90 | 45611.83 | 4.00 | 0.03 |
| + | + | + | + |  |  | + |  | + | + | 14 | -22792.07 | 45612.16 | 4.33 | 0.02 |

**Table S4.** Generalized linear model comparisons for nightly displacement (distance between 18:00h and 12:00h of the subsequent day) of raccoons translocated at the Savannah River Site (2018-2019). Levels of start (habitat where the animal was caught) and end (habitat where animal was moved) and are bottomland hardwood and upland pine. State refers to the translocation status and has 3 levels: control (pre-translocation), transient (exploratory post-translocation) and resident (stable post-translocation). Individual was included as a random effect. Model output includes sample size corrected Akaike’s information criterion (AIC_c_), Akaike weights (w_i_), log likelihood (LL), number of parameters (*K)*, and difference in AIC_c_ between each model and top model (ΔAIC_c_). Plus sign (+) in columns indicates the parameter was included in the model. Models with 10 lowest AIC_c_ values are presented.

| **End** | **State** | **Sex** | **Start** | **End:**  **State** | **End:**  **Sex** | **End:**  **Start** | **State:**  **Sex** | **State:**  **Start** | **Sex:**  **Start** | **df** | **logLik** | **AICc** | **delta** | **weight** |
| --- | --- | --- | --- | --- | --- | --- | --- | --- | --- | --- | --- | --- | --- | --- |
| + | + | + | + | + |  |  | + | + |  | 14 | -3345.52 | 6719.26 | 0.00 | 0.22 |
| + | + | + | + | + | + |  | + | + |  | 15 | -3345.00 | 6720.24 | 0.99 | 0.14 |
| + | + | + | + | + |  | + | + | + |  | 15 | -3345.23 | 6720.71 | 1.45 | 0.11 |
| + | + | + | + | + | + | + | + | + |  | 16 | -3344.45 | 6721.19 | 1.93 | 0.08 |
| + | + | + | + | + |  |  | + | + | + | 15 | -3345.49 | 6721.23 | 1.98 | 0.08 |
| + | + | + | + |  |  |  | + | + |  | 12 | -3349.02 | 6722.20 | 2.95 | 0.05 |
| + | + | + | + | + | + |  | + | + | + | 16 | -3344.97 | 6722.23 | 2.97 | 0.05 |
| + | + | + | + | + |  | + | + | + | + | 16 | -3345.22 | 6722.73 | 3.48 | 0.04 |
| + | + | + | + | + | + | + | + | + | + | 17 | -3344.27 | 6722.87 | 3.61 | 0.04 |
| + | + | + | + |  | + |  | + | + |  | 13 | -3348.62 | 6723.43 | 4.17 | 0.03 |

**Table S5.** Generalized linear model comparisons for distance between denning locations of raccoons translocated at the Savannah River Site (2018-2019). Levels of start (habitat where the animal was caught) and end (habitat where animal was moved) and are bottomland hardwood and upland pine. State refers to the translocation status and has 3 levels: control (pre-translocation), transient (exploratory post-translocation) and resident (stable post-translocation). Individual was included as a random effect. Model output includes sample size corrected Akaike’s information criterion (AIC_c_), Akaike weights (w_i_), log likelihood (LL), number of parameters (*K)*, and difference in AIC_c_ between each model and top model (ΔAIC_c_). Plus sign (+) in columns indicates the parameter was included in the model. Models with 10 lowest AIC_c_ values are presented.

| **End** | **Sex** | **Start** | **State** | **End:Sex** | **End:Start** | **End:State** | **Sex:Start** | **Sex:State** | **Start:State** | ***K*** | ***LL*** | **AIC_c_** | **ΔAIC_c_** | ***w_i_*** |
| --- | --- | --- | --- | --- | --- | --- | --- | --- | --- | --- | --- | --- | --- | --- |
| + | + | + | + |  |  |  |  | + | + | 12 | -2743.78 | 5511.78 | 0.00 | 0.14 |
| + | + | + | + |  |  |  |  |  | + | 10 | -2746.18 | 5512.52 | 0.75 | 0.10 |
| + | + | + | + |  |  |  | + | + | + | 13 | -2743.63 | 5513.52 | 1.74 | 0.06 |
| + | + | + | + |  | + |  |  | + | + | 13 | -2743.74 | 5513.73 | 1.96 | 0.05 |
| + | + | + | + | + |  |  |  | + | + | 13 | -2743.75 | 5513.76 | 1.98 | 0.05 |
| + |  | + | + |  |  |  |  |  | + | 9 | -2747.99 | 5514.11 | 2.34 | 0.04 |
|  | + | + | + |  |  |  |  | + | + | 11 | -2745.96 | 5514.12 | 2.34 | 0.04 |
| + | + | + | + |  |  | + |  | + | + | 14 | -2742.99 | 5514.28 | 2.51 | 0.04 |
| + | + | + | + |  |  |  | + |  | + | 11 | -2746.06 | 5514.31 | 2.53 | 0.04 |
| + | + | + | + |  | + |  |  |  | + | 11 | -2746.16 | 5514.51 | 2.73 | 0.04 |
